# A retrospective real‐world experience of immunotherapy in patients with extensive stage small‐cell lung cancer

**DOI:** 10.1002/cam4.5843

**Published:** 2023-07-18

**Authors:** Guihuan Qiu, Fei Wang, Xiaohong Xie, Ting Liu, Chen Zeng, Ziyao Chen, Maolin Zhou, Haiyi Deng, Yilin Yang, Xinqing Lin, Zhanhong Xie, Gengyun Sun, Chengzhi Zhou, Ming Liu

**Affiliations:** ^1^ Pulmonary and Critical Care Medicine, Guangzhou Institute of Respiratory Health, National Clinical Research Center for Respiratory Disease, National Center for Respiratory Medicine, State Key Laboratory of Respiratory Diseases The First Affiliated Hospital of Guangzhou Medical University Guangzhou China; ^2^ Department of Respiratory and Critical Care Medicine The First Affiliated Hospital of Anhui Medical University Hefei China

**Keywords:** PD‐1/PD‐L1 inhibitor, prognosis, real‐world study, safety, small‐cell lung cancer

## Abstract

**Background:**

The treatment of extensive stage small‐cell lung cancer (ES‐SCLC) has only made modest progress in the past decade, with two immune checkpoint inhibitors (ICIs), atezolizumab and durvalumab, approved for the treatment of SCLC by January 2022. However, currently, there is limited real‐world data on ES‐SCLC patients received immunotherapy.

**Methods:**

We retrospectively collected and analyzed the demographic and treatment data of ES‐SCLC patients at the First Affiliated Hospital of Guangzhou Medical University from January 2017 to January 2022. Survival and prognosis information was obtained through follow‐up.

**Results:**

A total of 353 ES‐SCLC patients were included, of which 165 received immunotherapy combined with chemotherapy as the first‐line (FL) treatment (chemo‐immune group), and 188 received chemotherapy (chemotherapy group). The objective response rate (ORR) and disease control rate (DCR) of patients receiving immunotherapy as the FL treatment were better than the chemotherapy group (76.97% vs. 48.40%, *p* < 0.001, and 83.03% vs. 68.09%, *p* < 0.001). Moreover, the progression‐free survival (PFS) and overall survival (OS) of ES‐SCLC patients receiving immunotherapy as the FL treatment were better than the chemotherapy group (6.7 months vs. 5.1 months, *p* < 0.001, and 12.5 months vs. 11.2 months, *p* < 0.001). Furthermore, the OS of ES‐SCLC patients who received immunotherapy as second‐line treatment was better than that in the chemotherapy group (15.9 months vs. 12.9 months, *p* = 0.036).

**Conclusion:**

ICIs combined with chemotherapy as the FL treatment could be beneficial to the ORR, DCR, PFS, and OS of ES‐SCLC patients. Furthermore, ES‐SCLC patients can benefit from ICIs in the second‐line treatment, even if they had not received ICIs in the FL treatment.

## Abstract 2

Small‐cell lung cancer is a subtype with a poor prognosis in lung cancer. Only two target PD‐L1 inhibitors have recently been approved in ES‐SCLC with moderate survival improvements based on clinical trials' results before 2022. Currently, there is relatively little data about the treatment patterns, efficacy, and safety of ES‐SCLC patients who received immunotherapy in the real world. This manuscript retrospectively collected and analyzed the demographic and treatment data of ES‐SCLC patients treated in the First Affiliated Hospital of Guangzhou Medical University from January 2017 to January 2022. We found that ICI combined with etoposide plus platinum (EP/C) as the FL treatment would prolong the PFS and OS in the ES‐SCLC. Chest radiotherapy with ICI administered may benefit the ES‐SCLC. ICI‐combined treatment in second/further‐line strategies might prolong the prognosis of the refractory SCLC even if patients did not receive ICI in the FL treatment. For the choice of ICIs in FL treatment for ES‐SCLC patients, PD‐1 inhibitor or PD‐L1 inhibitor cannot bring about significant differences in efficacy and safety.

## 1 INTRODUCTION

Small‐cell lung cancer (SCLC) is a refractory type of malignancy accounting for about 10%–15% of lung cancer cases. SCLC is characterized by rapid tumor cell proliferation, early distant metastasis, and poor prognosis.^1^, ^2^ Rubin et al. conducted a genetic study using RNA expression in SCLC cell lines derived from mice, dividing SCLC into four biological subtypes, SCLC‐A, SCLC‐N, SCLC‐P, and SCLC‐Y, based on different transcriptional regulatory factors, ASCL1, NEUROD1, POU2F3, and YAP1, respectively. However, the subsequent confirmatory experiment failed to confirm the SCLC‐Y subtype by immunohistochemical analysis.^3^ More recently, Gay et al. proposed a fourth subtype of SCLC‐inflammation (SCLC‐I), with no universal transcriptional features of ASCL1, NEUROD1, POU2F3, but the unique expression of genes that included many immune checkpoint markers and human leukocyte antigen (HLA).^4^ Moreover, data from Gay's study showed that SCLC‐I benefited the most from chemotherapy combined with immunotherapy.

In clinical practice, SCLC is classified as limited stage (LS), where lesions are limited to one rib cage, or extensive stage (ES), where the cancer spreads to the other lung, lymph nodes on the other side of the chest, or distant organs, according to the Veterans Administration Lung Study Group (VALSG).^5^ The VALSG staging system can be a guideline for treatment strategies and also serves as a predictor of prognosis for SCLC with no surgical opportunity. Approximately 70% of patients are ES at the time of diagnosis, which has a 5‐year survival rate <5%.^2^, ^6^ In the past decade, the first‐line (FL) standard treatment regimen for ES‐SCLC has been platinum‐containing doublet chemotherapy, while concurrent chemoradiotherapy is administrated for LS‐SCLC.^7^ Although the remission rate of SCLC in initial chemotherapy is 50%–70%, which can relapse within a few months, most cases of recurrent SCLC are not sensitive to the original regimen, and the median overall survival (OS) is generally 8–12 months.^2^, ^6^

The first immune checkpoint inhibitor (ICI), Ipilimumab (anti‐cytotoxic T lymphocyte‐associated antigen‐4), was approved for advanced melanoma in March 2011 based on the prognosis benefit in a phase III trial.^8^ Programmed cell death‐1 (PD‐1) inhibitors and programmed cell death ligand 1 (PD‐L1) inhibitors were subsequently administered in several solid tumors in recent years.^9^ The development of targeting the PD‐1/PD‐L1 pathway is a tremendous medical breakthrough in lung cancer therapy, especially in non‐small cell lung cancers (NSCLCs).^10^, ^11^ At the same time, SCLC ushered in a new treatment strategy change due to immunotherapy.^12^ Data from the phase III IMpower133 and CASPIAN trials showed the success of chemotherapy plus PD‐L1 inhibitors for patients with ES‐SCLC as the FL therapy option.^13^, ^14^ OS has improved for the first time in SCLC in recent decades, although only by 2 months. The Food and Drug Administration (FDA) and European Medicines Agency (EMA) had approved atezolizumab and durvalumab combined with platinum‐based doublet chemotherapy as the FL therapeutic strategy according to the clinical trials mentioned above.^15^ Following resistance of initial treatment in SCLC, back in August 2018, the FDA accelerated the approval of nivolumab and pembrolizumab as third‐line options for ES‐SCLC based on CheckMate‐032 and KEYNOTE‐158/028, respectively.^16^, ^17^ However, subsequent phase III clinical studies, Checkmate‐331/451 and KEYNOTE‐604, failed to confirm the benefit of these two ICIs in ES‐SCLC, resulting in FDA withdrawal of the indications in ES‐SCLC.

In summary, the current treatment strategies in SCLC are limited, and the clinical benefits are far less than those in NSCLC. Currently, most studies on the application of ICIs in SCLC are based on clinical trials. Therefore, more relevant data on treatment patterns, efficacy, and safety in the real world is required. In addition, there is a lack of research on the efficacy and safety of ICIs as a second‐line treatment for ES‐SCLC. Hence, we reviewed the data of 353 ES‐SCLC patients in our center over the past 5 years and explored the efficacy and prognosis of ICIs for ES‐SCLC patients.

## 2 PATIENTS AND METHODS

### 2.1 Patients

We retrospectively collected data on ES‐SCLC patients diagnosed and treated at the First Affiliated Hospital of Guangzhou Medical University from January 2017 to January 2022. The inclusion criteria were as follows: (I) defined pathological diagnosis of small‐cell lung cancer; (II) clinical stage of the patient is ES; (III) patients chose etoposide combined with platinum as FL chemotherapy and received at least one course of anti‐tumor therapy; and (IV) at least one measurable lesion with a single diameter (the minimum was not <10 mm, measured by spiral computed tomography [CT]). The exclusion criteria were as follows: (I) age < 18 years; and (II) incomplete demographic and treatment‐related data.

### 2.2 Data collection

We divided the ES‐SCLC patients into two cohorts, the chemo‐immune group, and the chemotherapy group, according to whether they received immunotherapy in the FL treatment. Data collected included: patients' demographics and baseline characteristics (gender, age, smoking status, and pack‐years, preexisting co‐morbidities, performance status, and metastatic sites at diagnosis); laboratory assessment of neutrophil granulocytes, eosinophils, lymphocytes, platelets, lactate dehydrogenase, neuron‐specific enolase, sodium, interleukin‐2 (IL‐2), IL‐4, IL‐6, IL‐10, tumor necrosis factor‐α (TNF‐α), and interferon‐γ (IFN‐γ) before treatment; treatment characteristics (radiation therapy, response at first courses, adverse events (AEs), and immune‐related adverse events [irAEs]); and follow‐up treatments.

The therapeutic effect index, including disease control rate (DCR) and objective response rate (ORR), was assessed using the Response Evaluation Criteria in Solid Tumors (RECIST) version 1.1. Progression‐free survival (PFS) was defined as the time from the initiation of therapy to disease progression or death. OS was defined as the time from initial treatment to the end; the last follow‐up was November 1, 2022.

The diagnostic criteria of SCLC were based on the “2015 Chinese Guidelines for the Diagnosis and Treatment of Primary Lung Cancer” and the tumor classification of patients followed the “World Health Organization Classification of Lung Tumor Tissue”.^18^, ^19^ The staging criteria are according to the VALSG.^5^

### 2.3 Statistical analysis

An unpaired t‐test was used to compare continuous variables between the observation and control groups. The Chi‐squared or Fisher's exact tests were used to compare categorical variables between the two groups. Univariate and multivariate analyses were performed using Cox proportional hazards models to investigate the risk factors associated with fatal outcomes. Risk factors with *p* ≤ 0.1 in the univariate analysis were included in the multivariate analysis. The hazard ratio (HR) was reported along with the 95% confidence interval (CI). Kaplan–Meier survival curves were used to evaluate the PFS and OS of patients, and the log‐rank test was used to assess differences between groups. A *p*‐value of <0.05 indicated statistical significance for all tests. The statistical significance levels were all two‐sided. SPSS version 22.0 was used for analysis, and the graphs were generated using GraphPad Prism 8.0.2.

### 2.4 Ethics statement

Institutional review board/ethics committee approval was obtained from the Institutional Review Board of the First Affiliated Hospital of Guangzhou Medical University (No. 2020‐189, 2021‐1‐13), which was then approved by each participating center. Furthermore, the research procedures followed the ethical standards of the responsible committee on human experimentation and were in accordance with the Helsinki Declaration of 1975.

## 3 RESULTS

### 3.1 Baseline characteristics of ES‐SCLC patients

We included a total of 353 ES‐SCLC patients, of which 188 chose etoposide combined with platinum as the FL chemotherapy regimen for anti‐tumor treatment (chemotherapy group), and the remaining 165 chose immunotherapy combined with chemotherapy as the first‐line treatment (chemo‐immune group). ICIs for patients included sintilimab, camrelizumab, toripalizumab, tislelizumab, nivolumab, pembrolizumab (PD‐1 inhibitors); and atezolizumab and durvalumab (PD‐L1 inhibitors). Twenty‐two patients in the chemo‐immune group received two courses of induction chemotherapy before immunotherapy. The procedure for screening eligible cases is shown in Figure S1. The enrolled ES‐SCLC patients were mainly men with a smoking history, most of whom had central‐type lung cancer. Most patients had a performance status (PS) score of 0–1 (Table 1). Compared with the chemotherapy group, there were more patients aged >65 years and smokers in the chemo‐immune group. In addition, more patients in the chemo‐immune group had already developed intrapulmonary, liver, and adrenal metastases at the time of diagnosis. Compared to the values of the laboratory examination between the patients in the two study cohorts, patients in the chemo‐immune group had higher serum sodium (137.65 vs. 136.30 mmol/L, *p* = 0.008).

**TABLE 1:** Differences in clinical characteristics between the ES‐SCLC patients with chemo‐immunotherapy and chemotherapy as first line.

Category and Sub‐category	Total patients (*n* = 353)	Chemo‐immune (*n* = 165)	Chemotherapy (*n* = 188)	*p* value
Age (year) mean ± SD	62.40 ± 8.59	63.80 ± 8.83	61.26 ± 8.23	0.005**
Age (year), ≥65, *n* (%)	163 (46.18)	89 (53.94)	74 (39.36)	0.006**
Gender, Male, *n* (%)	330 (93.48)	151 (91.52)	179 (95.21)	0.113
Smoking history, *n* (%)	253 (71.67)	105 (63.64)	148 (78.72)	0.002**
PS scores, 0–1, *n* (%)	305 (86.40)	137 (83.03)	168 (89.36)	0.083
Location, Central, *n* (%)	265 (75.07)	121 (73.33)	144 (76.60)	0.480
Metastatic sites, *n* (%)				
Intrapulmonary	62 (17.56)	39 (23.64)	23 (12.23)	0.005**
Liver	94 (26.63)	56 (33.94)	38 (20.21)	0.004**
Brain	81 (22.95)	56 (33.94)	25 (13.30)	0.761
Bone	107 (30.31)	65 (39.39)	42 (22.34)	0.215
Adrenal	69 (19.55)	50 (30.30)	19 (10.11)	0.001**
Distinct lymph nodes	35 (9.92)	16 (9.70)	19 (10.11)	0.898
Pleura	130 (36.83)	66 (40.00)	64 (34.04)	0.437
Other	34 (9.63)	15 (9.09)	19 (10.11)	0.247
Anti‐vascular therapy, *n* (%)	29 (8.22)	15 (9.09)	14 (7.45)	0.575
Thoracic radiotherapy, *n* (%)	75 (21.25)	39 (23.64)	36 (19.15)	0.318
Brain radiotherapy, *n* (%)	21 (5.95)	11 (6.67)	10 (5.32)	0.593
COPD, *n* (%)	151 (42.78)	70 (42.42)	81 (43.09)	0.900
Interstitial pneumonia, *n* (%)	15 (4.25)	6 (3.64)	9 (4.79)	0.593
Laboratory examination (median [IQR])				
Neutrophil granulocyte	5.20 (4.00, 6.90)	5.40 (4.30, 6.90)	5.10 (3.80, 6.90)	0.102
Lymphocyte	1.60 (1.20, 2.00)	1.60 (1.20, 2.00)	1.60 (1.10, 2.00)	0.340
Eosinophils	0.12 (0.10, 0.22)	0.11 (0.10,0.26)	0.12 (0.09, 0.20)	0.430
Platelet	278.0 (220.8, 340.3)	282.0 (224.0, 342.8)	272.0 (208.8, 331.0)	0.644
Lactate dehydrogenase	239.5 (193.8, 327.7)	240.5 (201.8, 337.2)	236.5 (179.5, 328.3)	0.254
Neuron specific enolase	59.36 (31.96,117.85)	64.42 (36.34, 112.20)	51.97 (27.45,106.20)	0.05
Serum sodium	137.00 (133.80, 139.40)	137.65 (134.50,139.63)	136.30 (132.65, 139.10)	0.008**
IL‐2		1.39 (0.71, 1.97)		
IL‐4		1.75 (0.96, 2.57)		
IL‐6		8.84 (3.91, 19.34)		
IL‐10		2.86 (2.13, 3.96)		
TNF‐α		1.62 (0.82, 3.03)		
IFN‐γ		1.49 (1.07, 2.35)		

*Note*: Range of normal values: Neutrophil granulocyte (1.8–8.0*10^9^/L), Eosinophils (0.05–0.3*10^9^/L), Lymphocyte (0.90–5.20*10^9^/L), Platelet (100–400*10^9^/L), Lactate dehydrogenase (109–225 U/L), Neuron specific enolase (0.00–16.3 ng/mL), serum sodium (135–145 mmol/L), IL‐2 (0–5.71 pg/mL), IL‐4 (0–2.8 pg/mL), IL‐6 (0–5.30 pg/mL), IL‐10 (0–4.91 pg/mL), TNF‐α (0–4.60 pg/mL), IFN‐γ (0–7.42 pg/mL). Abbreviations: COPD, chronic obstructive pulmonary disease; ECOG PS, Eastern cooperative oncology group performance status; IQR, intra quartile range; SD, standard deviation.

** *p* < 0.01.

### 3.2 Treatment patterns in ES‐SCLC patients

We compared the response at the first evaluation of the two groups. We found that the ORR and DCR of 165 patients who chose FL immunotherapy combined with chemotherapy were 76.97% and 83.03%, respectively, which were significantly better than in the chemotherapy group (48.40% and 68.09%, respectively; *p* < 0.001). Regarding the selection of ICIs in ES‐SCLC patients in the chemo‐immune group, 91 (55.15%) patients chose PD‐1 inhibitors, while the remaining 77 (44.85%) patients chose PD‐L1 inhibitors. The course of chemotherapy as the FL treatment in the chemo‐immune group was longer than that in the chemotherapy group (4.6 ± 2.1 times vs. 3.8 ± 2.1 times, *p* < 0.001). In addition, 12.73% of patients in the chemo‐immune group had irAEs; immunotherapy‐related hypothyroidism occurred the most frequently (4.85%), and 3.03% of patients developed hypothyroidism, 1.21% of patients developed dermatitis, 1.21% of patients developed enteritis, 0.61% of patients developed immunotherapy‐related myocarditis, and 0.61% of patients developed immunotherapy‐related thrombocytopenia. By the end of the follow‐up, 117 patients in the chemo‐immune group had tumor progression, and 61.54% had received second‐line treatment. Of the 145 patients in the chemotherapy group who developed tumor progression, 55.86% received second‐line therapy, and 31 chose immunotherapy monotherapy or a combination with chemotherapy as second‐line treatment (Table 2).

**TABLE 2:** Treatment characteristics of ES‐SCLC patients in two groups.

Category and Sub‐category	Chemo‐immune (*n* = 165)	Chemotherapy (*n* = 188)	*p* value
Response at the first evaluation			
CR	5	2	
PR	112	89	
SD	19	42	
PD	10	37	
Not assessable	19	18	
ORR (%)	80.14	53.53	<0.001***
DCR (%)	93.15	78.24	<0.001***
AEs (3–4), *n* (%)	18 (10.91)	13 (6.91)	0.186
Bone marrow suppression	14 (8.48)	8 (4.26)	
Radiation pneumonitis	4 (2.42)	5 (2.66)	
irAE (3–4), *n* (%)	21 (12.73)	‐	
Hypothyroidism	5		
Pneumonia	8		
Enteritis	2		
Dermatitis	2		
Hepatitis	2		
Myocarditis	1		
Thrombocytopenia	1		
ICIs, *n* (%)			
PD‐1	91 (55.15)		
PD‐L1	74 (44.85)		
The courses of first‐line treatment (mean, SD)			
Chemotherapy	4.59 ± 2.09	3.76 ± 2.13	0.000***
Immunotherapy	6.09 ± 4.93		
Patients with tumor progression, *n* (%)	117 (70.91)	145 (77.13)	
Receiving second‐line treatment, *n* (%)	72 (61.54)	81 (55.86)	0.354
Immunotherapy as second‐line treatment	47	31	

Abbreviations: AEs, adverse events; CR, complete response; DCR, disease control rate; ICIs, immune checkpoint inhibitors; irAEs, immune‐related adverse events; ORR, objective response rate; PD, progressive disease; PR, partial response; SD, stable disease.

*** *p* < 0.001.

### 3.3 Prognosis and survival analysis of ES‐SCLC patients

**FIGURE 1:**
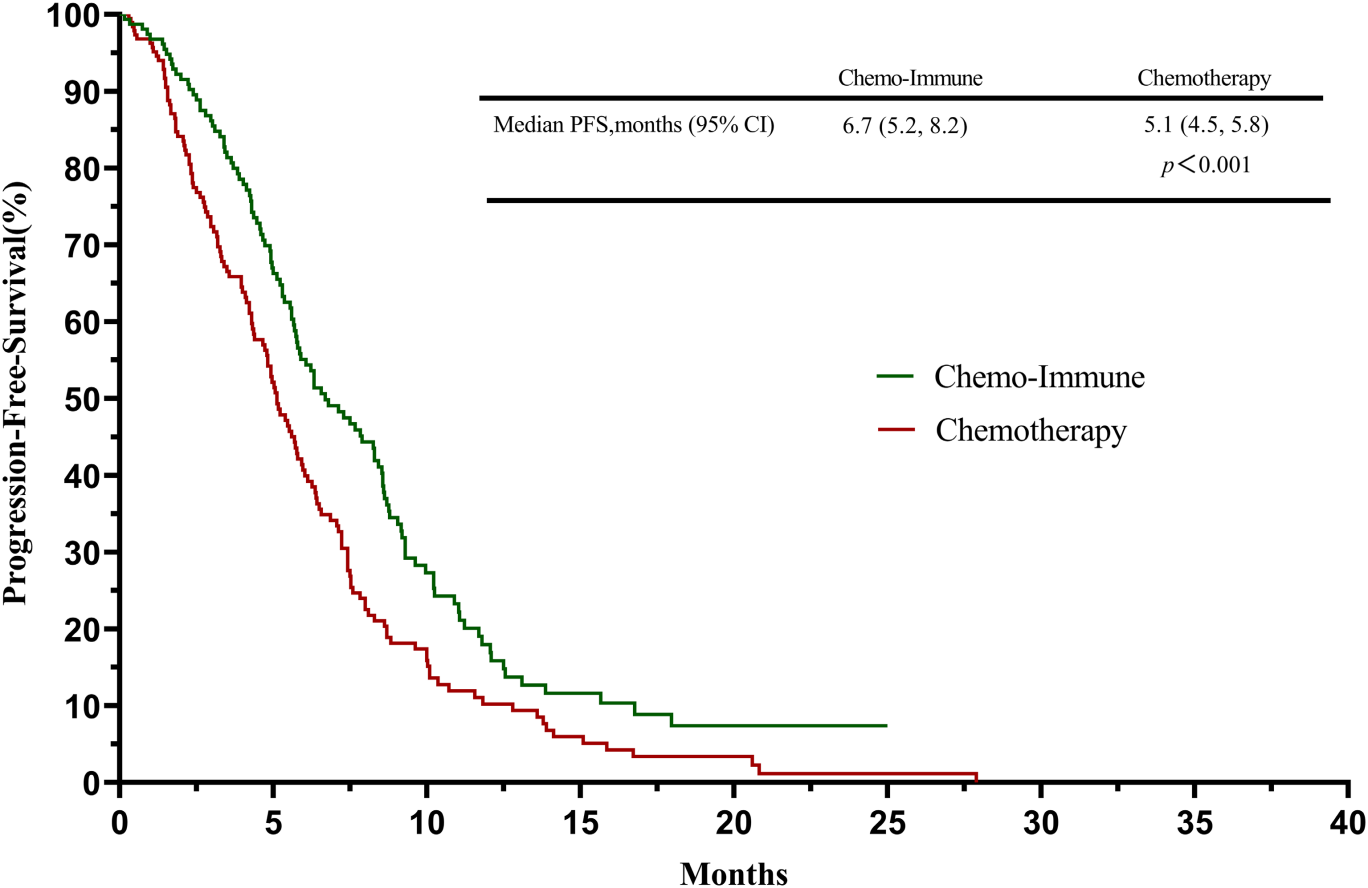
Survival curves of progression‐free survival (PFS) between extensive stage small‐cell lung cancer (ES‐SCLC) patients who chose immunotherapy combined with chemotherapy and chemotherapy as the first‐line (FL) treatment.

**FIGURE 2:**
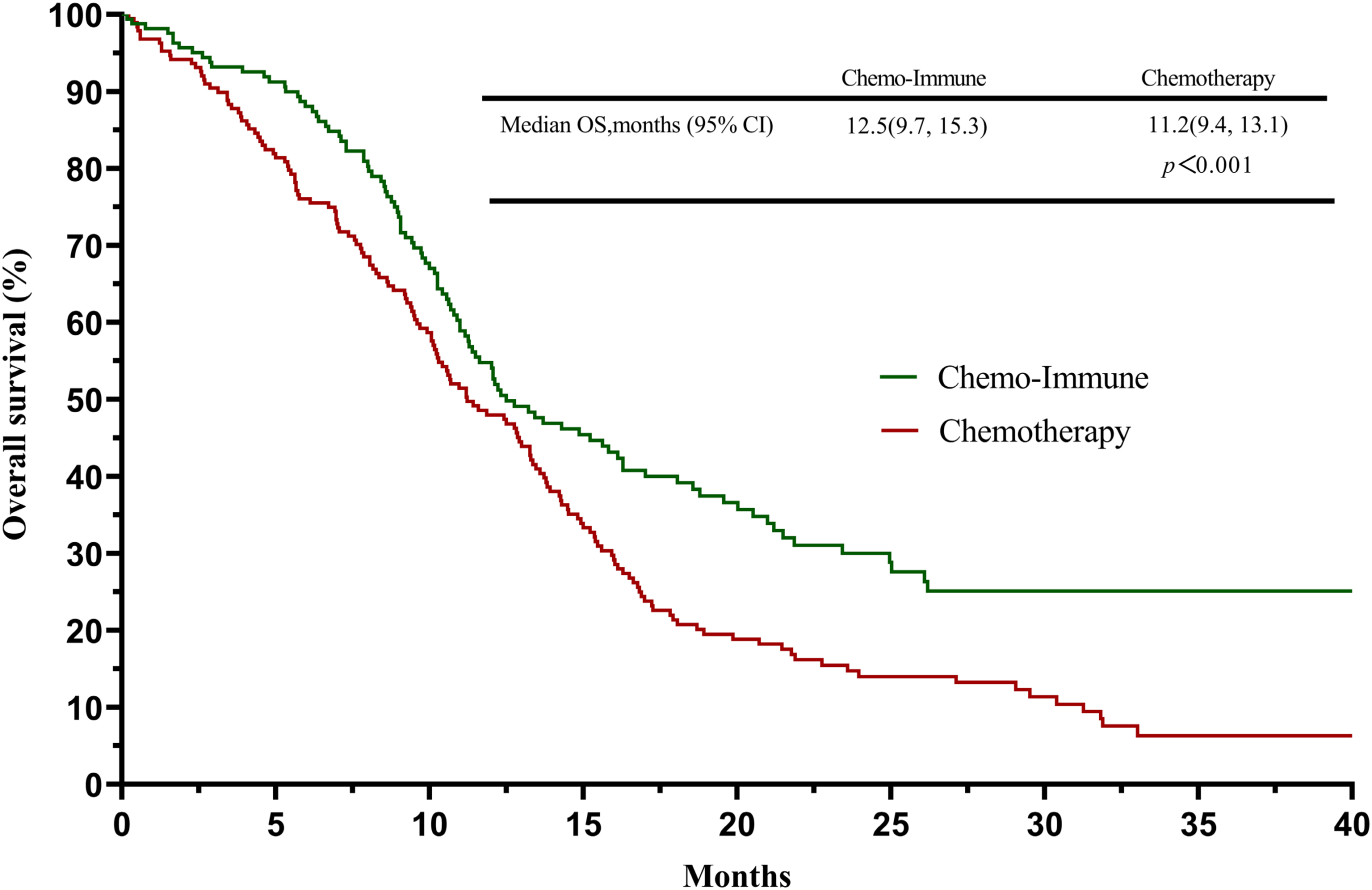
Survival curves of overall survival (OS) between extensive stage small‐cell lung cancer (ES‐SCLC) patients who chose immunotherapy combined with chemotherapy and chemotherapy as the first‐line (FL) treatment.

By the endpoint of the follow‐up, 105 ES‐SCLC patients had died in the chemo‐immune group and 162 in the chemotherapy group. Based on the follow‐up information, we plotted the survival curves of PFS and OS for both groups (Figures 1 and 2). The PFS of patients in the chemo‐immune group was better than the chemotherapy group (6.7 months vs. 5.1 months, *p* < 0.001), and the OS of the chemo‐immune group was better than the chemotherapy group (12.5 months vs. 11.2 months, *p* < 0.001). After the multivariate and univariate analysis of the 353 ES‐SCLC patients, we found that in addition to FL immunotherapy, poor PS score (≥2) at diagnosis and no thoracic radiotherapy were independent risk factors for poor survival and prognosis (Table 3; Figure S2).

**TABLE 3:** Univariate and multivariate analysis of ES‐SCLC patients on overall survival by Cox proportional hazard regression model.

Category and Sub‐category	Univariate analysis		Multivariate analysis	
	HR (95% CI)	*p* value	HR (95% CI)	*p* value
Age, years				
≥65 vs. <65	0.91 (0.71, 1.16)	0.441	‐	‐
Gender				
Male vs. Female	1.66 (1.22, 2.25)	0.001	1.34 (0.94, 1.89)	0.104
Smoking history				
No vs. Yes	0.92 (0.33, 2.51)	0.863	‐	‐
PS scores				
0–1 vs. ≥2	1.84 (1.31, 2.57)	0.000	1.59 (1.09, 2.33)	0.016*
Location				
Central vs. Peripheral	0.98 (0.75, 1.29)	0.907	‐	‐
Metastatic sites (No vs. Yes)				
Intrapulmonary	1.24 (0.83, 1.85)	0.289	‐	‐
Liver	1.29 (0.96, 1.74)	0.089	1.05 (0.77, 1.44)	0.761
Brain	1.40 (1.07, 1.84)	0.014	1.20 (0.90, 1.59)	0.215
Bone	1.29 (0.98, 1.69)	0.071	‐	‐
Adrenal	0.95 (0.67, 1.35)	0.759	‐	‐
Distinct lymph nodes	1.46 (0.99, 2.15)	0.055	1.18 (0.78, 1.77)	0.437
Pleura	0.93 (0.72, 1.19)	0.544	‐	‐
Other	1.36 (0.92, 2.00)	0.120	‐	‐
ICIs				
No vs. Yes	0.65 (0.51,0.83)	0.001	0.74 (0.56,0.99)	0.039*
Anti‐vascular				
No vs. Yes	0.88 (0.57,1.36)	0.553	‐	‐
Thoracic radiotherapy				
No vs. Yes	0.62 (0.46,0.85)	0.003	0.72 (0.52,0.99)	0.045*
Brain radiotherapy				
No vs. Yes	0.79 (0.49,1.27)	0.322	‐	‐
Co‐morbidities (No vs. Yes)				
COPD	1.12 (0.88,1.44)	0.349	1.13 (0.47,2.74)	0.401
Interstitial pneumonia	1.25 (0.70,2.22)	0.458	‐	‐

Abbreviations: HR, hazard rate; PS, performance status; ICIs, immune checkpoint inhibitors; COPD, chronic obstructive pulmonary disease.

* *p* < 0.05

### 3.4 Subgroup analysis between ES‐SCLC patients with PD‐1 inhibitors and PD‐L1 inhibitors

We divided the 165 ES‐SCLC patients in the chemo‐immune group into the PD‐1 subgroup (*n* = 91) and PD‐L1 subgroup (*n* = 74) according to the choice of ICIs. Treatment characteristics and prognosis of patients between PD‐1 and PD‐L1 subgroups are shown in Table S1, Figures S3 and S4. The results showed no statistical difference in the treatment efficacy and survival prognosis between the two groups of patients (PD‐1subgroup vs.PD‐L1 subgroup: PFS, 8.30 months vs. 5.70 months, *p* = 0.080; OS, 14.30 months vs. 12.33 months, *p* = 0.893). In addition, there was no statistical difference in the occurrence of treatment‐related AEs (including irAEs) between PD‐1 and PD‐L1 subgroups.

### 3.5 Subgroup analysis of ES‐SCLC patients in second‐line treatment

In the chemo‐immune group, 72 patients received second‐line treatment after tumor progression. Forty‐seven chose immunotherapy as second‐line treatment, and their OS was not significantly different from other patients (13.4 months vs. 12.0 months, *p* = 0.609). The survival curve is shown in Figure S5.

**FIGURE 3:**
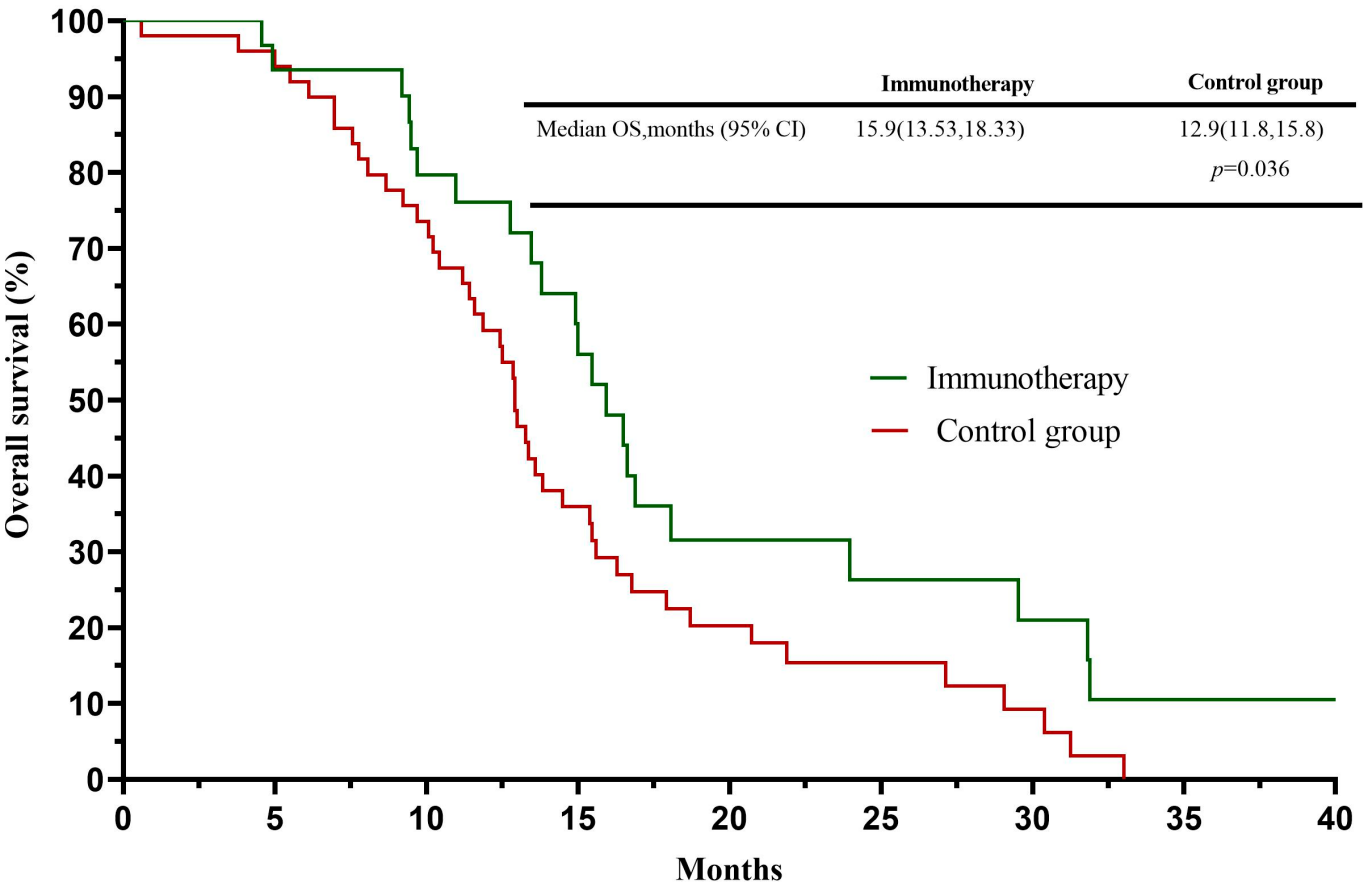
Survival curves of subgroups between patients who chose immunotherapy and no‐immunotherapy as the second‐line treatment in the chemotherapy group.

In the chemotherapy group, 81 patients received second‐line treatment after tumor progression. Furthermore, we selected 31 patients who received immunotherapy in the second‐line treatment as the immunotherapy group and the remaining 50 as the control group for subgroup analysis. The characteristics of patients between the immunotherapy group and control groups are shown in Table S2. We found that the survival time of patients in the immune group was better than that in the control group (15.9 months vs. 12.9 months, *p* = 0.036); the survival curve is shown in Figure 3.

## 4 DISCUSSION

A high tumor mutation burden (TMB) and tumor cell programmed death ligand (PD‐L1) expression are associated with immunotherapy response.^20^, ^21^ Studies have confirmed that the occurrence of SCLC is significantly related to tobacco consumption, and long‐term tobacco exposure can lead to an increase in the TMB.^22^ Theoretically, SCLC is sensitive to initial chemotherapy, which can promote the release of immune antigens and increase the T cell response. In addition, the high frequency of somatic mutations caused by long‐term exposure to carcinogens in cigarettes, and the presence of paraneoplastic syndromes showing an anti‐Hu autoimmune response, suggest that SCLC may benefit from immunotherapy. Together, this provides a theoretical basis for combining immunotherapy and chemotherapy in SCLC.

The IMpower133 clinical trial is an international, double‐blind, randomized, placebo‐controlled phase III trial, which evaluated the efficacy and safety of FL treatment with etoposide plus carboplatin in combination with atezolizumab (anti‐PD‐L1 inhibitor) compared to chemotherapy combined with placebo in treatment‐naive ES‐SCLC. A total of 403 de novo diagnoses of ES‐SCLC were enrolled in the study. The median OS (12.3 months vs. 10.3 months, HR: 0.70, 95% CI: 0.54–0.91, *p* = 0.007), PFS (5.2 months vs. 4.3 months; HR: 0.77, 95% CI: 0.62–0.96, *p* = 0.02) were improved, but there was no difference in the ORR.^13^ Therefore, the FDA approved atezolizumab in combination with chemotherapy as a FL treatment for patients with ES‐SCLC on March 18, 2019. In 2019, the CASPIAN trial further validated the success of FL chemotherapy combined with immunotherapy as a treatment strategy for patients with ES‐SCLC.^14^ The two clinical trials mentioned above showed an improved median OS in ES‐SCLCs with immunotherapy, which became a historic breakthrough. However, in contrast to the promising outcomes in NSCLC, the benefit of immunotherapy in SCLC patients is limited, and treatment strategies available for ES‐SCLC are scarce. Therefore, there is an urgent need for innovative immunotherapy to benefit patients with ES‐SCLC. Recently, the CAPSTONE‐1 study showed that adebrelimab (a novel anti‐PD‐L1 inhibitor) combined with chemotherapy significantly prolonged the OS in ES‐SCLC with a tolerable safety profile.^23^ Most recently, the ASTRUM‐005 study confirmed that the combination of serplulimab based on FL standard chemotherapy brought comprehensive benefits in the OS, PFS, ORR, and duration of response (DoR) to patients with ES‐SCLC, achieving a breakthrough in this field of anti‐PD‐1 inhibitors, and confirmed, for the first time, that PD‐1 inhibitor combined with chemotherapy could also improve the survival of ES‐SCLC patients.^24^ This further suggests that the application of ICIs could enable a more favorable prognosis in SCLC.

The ages of the patients in the present study were not significantly different from the previous two studies (CASPIAN study: 63, 62, and 63 years old; IMpower133 study: 64 and 64 years old; our study: 63.80 ± 8.83 years and 61.26 ± 8.23 years). However, the proportion of male patients was higher (CASPIAN study: 75%, 71%, and 68%; IMpower133 study: 64.2% and 65.3%; our study: 91.52% and 95.21%), with a lower proportion of smokers (CASPIAN study: 95%, 92%, and 94%; IMpower133 study: 91% and 97%; our study: 63.64% and 78.72%), and there was a higher frequency of brain metastases (CASPIAN study: 14%, 10%, and 10%; IMpower133 study: 8.5% and 8.9%; our study: 33.94% and 13.30%) in our study. In addition, compared with the previous two randomized controlled trials, our cohort included some patients with a PS score (≥2), which is the most significant difference. Furthermore, in the present retrospective real‐world study, the PFS of 5.1 months was better than that reported in the previous IMpower133 and CASPIAN trials. The OS of patients who received ICIs was consistent with that of the previous studies mentioned above, while the OS of 11.2 months in the chemotherapy alone as the FL treatment was better than the previous two trials. A better PS has been considered as an indicator related to prolonged prognosis in previous retrospective real‐world studies.^25^, ^26^, ^27^, ^28^

In the multivariate and univariate analysis of the present study, a poor PS score (≥2) at diagnosis was found to be an independent risk factor for worse survival and prognosis. Although a certain number of poor PS score (2, 3) patients were included, the OS of the patients in this study was not worse than that in the above two studies. In our chemotherapy group, 55.86% of patients received second or further line treatment strategies, and 31 of them were administered ICIs; the survival time of these patients with ICIs was still better than patients without ICIs (15.9 months vs. 12.9 months, *p* = 0.036). A previous study has shown that ICI‐combination patterns in the second‐line treatment can still be beneficial for recurrent SCLCs.^28^ Therefore, ES‐SCLC patients can benefit from ICIs in the second‐line treatment even if they had not received ICIs in the FL treatment.

A meta‐analysis study of 2905 ES‐SCLCs, which included six previous studies, identified that the addition of PD‐1/PD‐L1 inhibitors to chemotherapy could result in favorable outcomes regarding the PFS and OS in ES‐SCLC FL treatment settings without additional AEs.^29^ Recently, a retrospective study showed that PD‐1/PD‐L1 inhibitors in a combined pattern of treatment strategies might improve the prognosis of SCLC.^28^ In our study, the ORR, DCR, PFS, and OS of patients in the chemo‐immune group were significantly better than those in the chemotherapy group. In addition, 55.15% of patients who received PD‐1inhibitors and 44.85% who received PD‐L1 inhibitors were included in the ICI group; however, there were no significant difference in the ORR, DCR, PFS, and OS between the PD‐1 group and PD‐L1 group. Combined with the dramatic clinical outcomes in the ASTRUM‐005 study,^24^ the addition of a PD‐1 inhibitor could benefit SCLC patients. Prospective studies should be conducted to explore the effect and safety of different PD‐1inhibitors in ES‐SCLC. In this multivariate and univariate analysis conducted on 353 patients, we found that ICIs in FL treatment, better PS (0–1) at diagnosis, and thoracic radiotherapy were related to better survival and prognosis in ES‐SCLC. Concurrent or sequential chemoradiotherapy settings have significantly improved the prognosis in SCLC.^30^, ^31^, ^32^ Among the patients who chose immunotherapy combined with chemotherapy as the FL treatment, 12.73% had irAEs (grade 3–4), which were mainly pneumonia and hypothyroidism. Compared with previous prospective studies (IMpower133, etc.), the frequency of AEs in our study is fewer. The reason for this difference may be the omission of relevant adverse reaction information due to the limitations of retrospective studies.

This is a real‐world study on the efficacy and safety of PD‐1/PD‐L1 inhibitors in 353 ES‐SCLC patients. Compared with previous clinical trials, the results of this study can better reflect the clinical practice application of ICIs in ES‐SCLC patients. For example, the patients in our cohort included some patients with poor PS scores (2, 3), which were not provided in previous clinical trials. Therefore, to the best of our knowledge, this is the first study to comprehensively analyze the efficacy and safety of both PD‐1 inhibitors and PD‐L1 inhibitors combined with chemotherapy as the FL treatment in ES‐SCLC in the real world; we found that the prognosis of patients treated with PD‐1 inhibitors is not worse than patients treated with PD‐L1 inhibitors.

There are some limitations in the current study. (1) As a retrospective study relying on electronic medical records and telephone follow‐up, the subjects included in this study were enrolled over a long period (the earliest time of treatment among enrolled patients was January 2017), thus leading to the assessment of inconsistent outcomes for the patient. (2) We had not recorded some important biomarkers currently considered effective predictors of immunotherapy, such as PD‐L1 and TMB; these may be critical factors for the efficacy and prognosis of patients in our study. (3) There are omissions in the records of treatment‐related AEs, mainly caused by doctors' lack of knowledge of irAEs for early immunotherapy cases; this critical information could not be completed in the follow‐up. (4) This is a real‐world study that enrolled patients over 5 years, thus, the comparison of baseline data of the two cohorts has limitations, including sex, age, and tumor metastasis.

## 5 CONCLUSIONS

The present study showed that ICI combined with etoposide and cisplatin chemotherapy as the FL treatment could prolong the PFS and OS of ES‐SCLC patients. Thoracic radiotherapy with ICI administration might be beneficial in ES‐SCLC; however, more prospective studies were required to explore the safety and efficacy of radiotherapy administered during immunotherapy. ICI‐combined treatment in second/further ‐line strategies might prolong the prognosis of refractory SCLC, even if patients did not receive ICI in the FL treatment. Regarding the choice of ICIs in the FL treatment for ES‐SCLC patients, PD‐1 inhibitors or PD‐L1 inhibitors did not show significant differences in efficacy and safety.

## AUTHOR CONTRIBUTIONS

**Guihuan Qiu:** Conceptualization (lead); formal analysis (lead); methodology (equal); writing – original draft (lead). **Fei Wang:** Conceptualization (equal); data curation (equal); formal analysis (lead); writing – original draft (lead). **Xiaohong Xie:** Conceptualization (equal); data curation (equal); investigation (lead); writing – original draft (supporting). **Ting Liu:** Data curation (supporting); formal analysis (supporting); investigation (lead); methodology (supporting); resources (supporting). **Chen Zeng:** Methodology (lead); software (supporting); validation (supporting); visualization (supporting). **Ziyao Chen:** Data curation (supporting); investigation (equal); resources (lead); validation (supporting); visualization (supporting). **Maolin Zhou:** Methodology (equal); resources (lead); software (equal). **Haiyi Deng:** Methodology (lead); resources (equal); software (supporting); supervision (supporting). **Yilin Yang:** Funding acquisition (equal); investigation (supporting); software (lead); supervision (supporting). **Xinqing Lin:** Investigation (equal); visualization (supporting); writing – original draft (supporting). **Zhanhong Xie:** Resources (supporting); supervision (supporting); validation (supporting); visualization (supporting). **Gengyun Sun:** Formal analysis (supporting); resources (supporting); validation (equal); visualization (supporting). **Chengzhi Zhou:** Conceptualization (supporting); funding acquisition (lead); project administration (lead); writing – original draft (supporting); writing – review and editing (equal). **Ming Liu:** Conceptualization (supporting); funding acquisition (equal); resources (lead); visualization (lead); writing – original draft (equal); writing – review and editing (lead).

## CONFLICT OF INTEREST STATEMENT

The authors declare that the research was conducted in the absence of any commercial or financial relationships that could be construed as a potential conflict of interest.

## FUNDING STATEMENT

This study was supported by grants from Chinese Society of Clinical Oncology [Y‐HS202102‐0118]; State Key Laboratory of Respiratory Disease‐The Independent project [2020GIRH007]; Zhijiang Laboratory‐The open project [2021PE0AC06].

## ETHICS APPROVAL STATEMENT

This study was approved by the Ethics Committee of The First Affiliated Hospital of Guangzhou Medical University (Guangzhou, Guangdong, China, No. 2020–189, 2021‐1‐13). Study was performed in accordance with the ethical standards of the Declaration of Helsinki. There was no additional invasive test or experimental drugs used out of order for the patients.

## PATIENT CONSENT STATEMENT

The patients/participants provided their written informed consent to participate in this study.

## PERMISSION TO REPRODUCE MATERIAL FROM OTHER SOURCES

NA.

## CLINICAL TRIAL REGISTRATION

NA.

## Supporting information


Figure S1.
Figure S2.
Figure S3.
Figure S3.
Figure S4.
Figure S5.

Table S1.
Table S2.

## Data Availability

Data presented in this study are included in the article/supplementary materials. Further data accessed should via email the authors.
