# Supplementary material for: A retrospective real‐world experience of immunotherapy in patients with extensive stage small‐cell lung cancer
Source: Cancer Med. 2023 Jul 18;12(14):14881–91. doi: 10.1002/cam4.5843 (PMC10417203; doi:10.1002/cam4.5843)
Supplement: Supplementary file 1 — Figure S1. Figure S2. Figure S3. Figure S3. Figure S4. Figure S5. [file CAM4-12-14881-s002.docx]

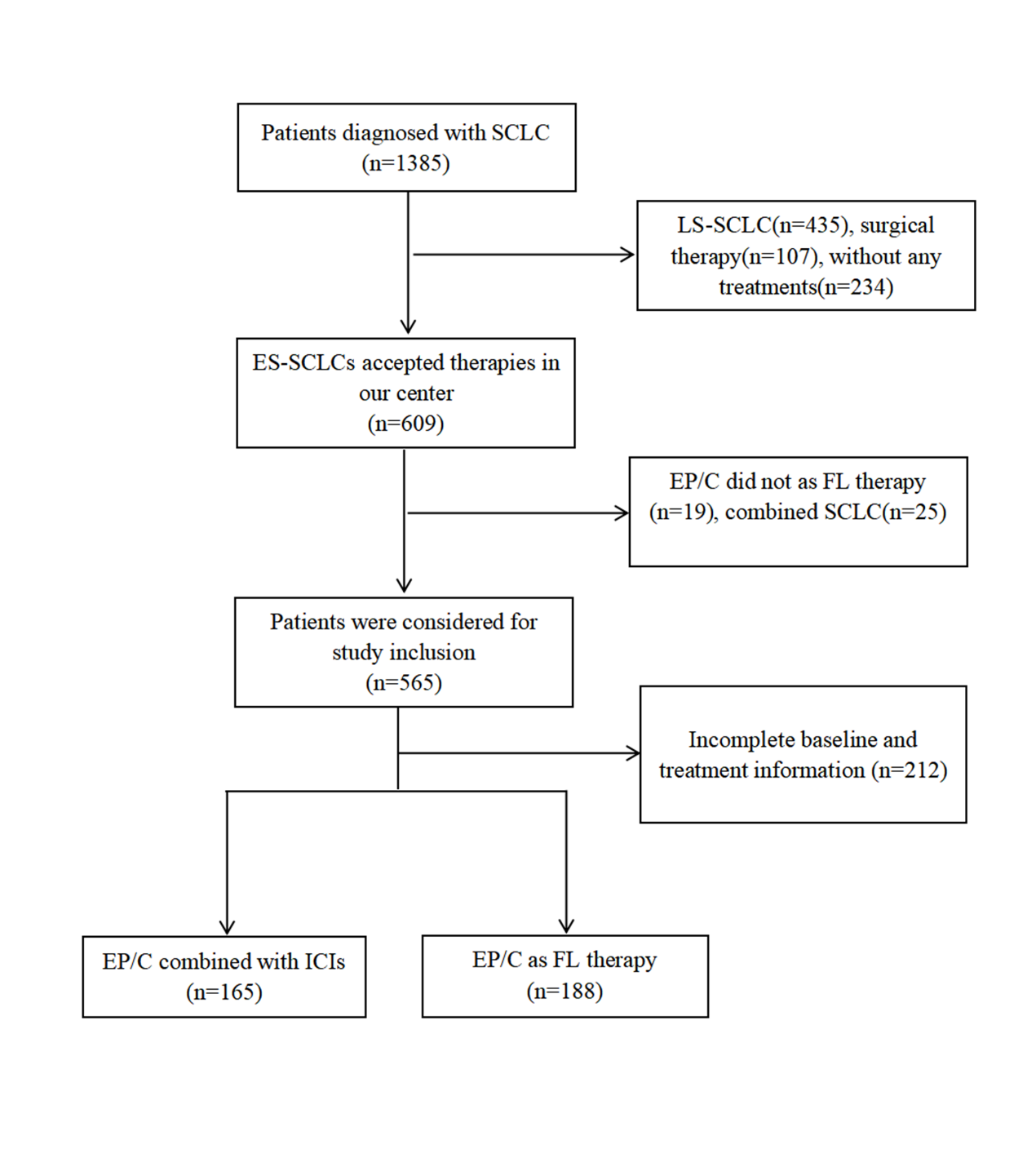


**Supplemental Figure 1.** The flow chart of study design and patient inclusion. SCLC-small cell lung cancer, LS-SCLC-limited stage small cell lung cancer, ES-SCLC-extensive stage small cell lung cancer, EP/C-etoposide platinum/carboplatin，FL-first line, ICIs-immune checkpoint inhibitors.


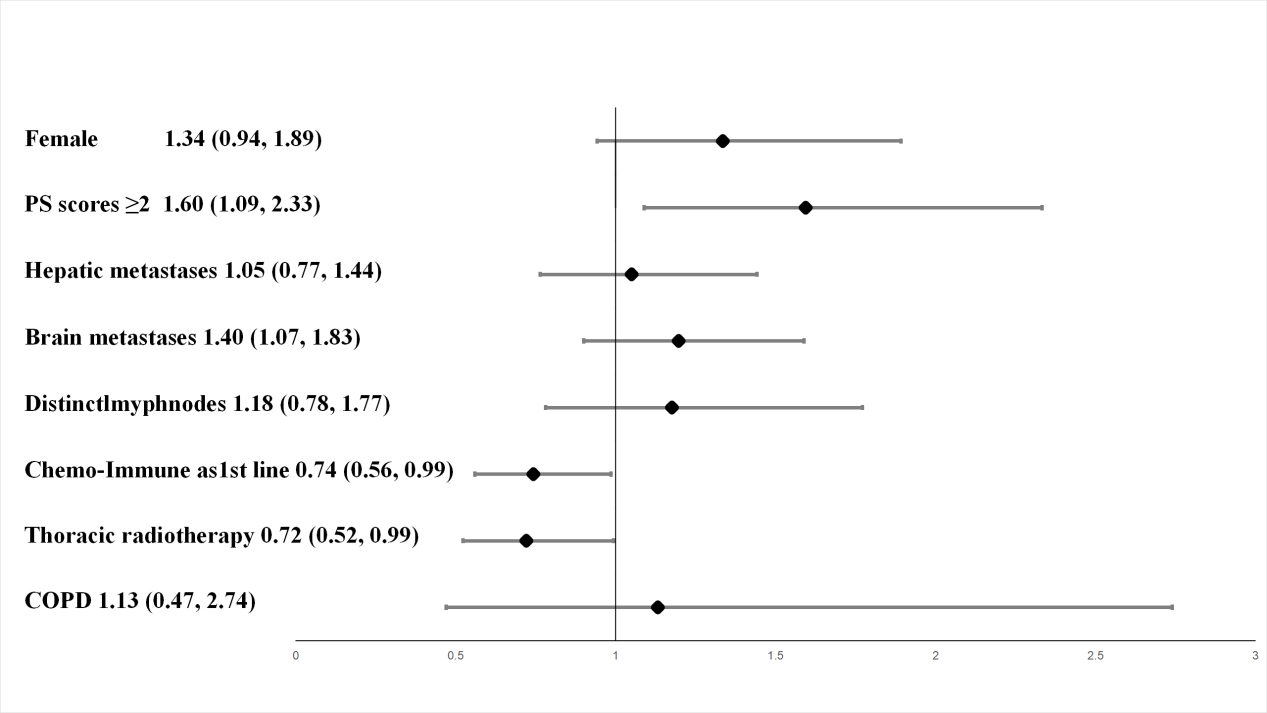


**Supplemental Figure 2.** The forest plot for multi-factorial regression analysis of patients with extensive stage small cell lung cancer (ES-SCLC).


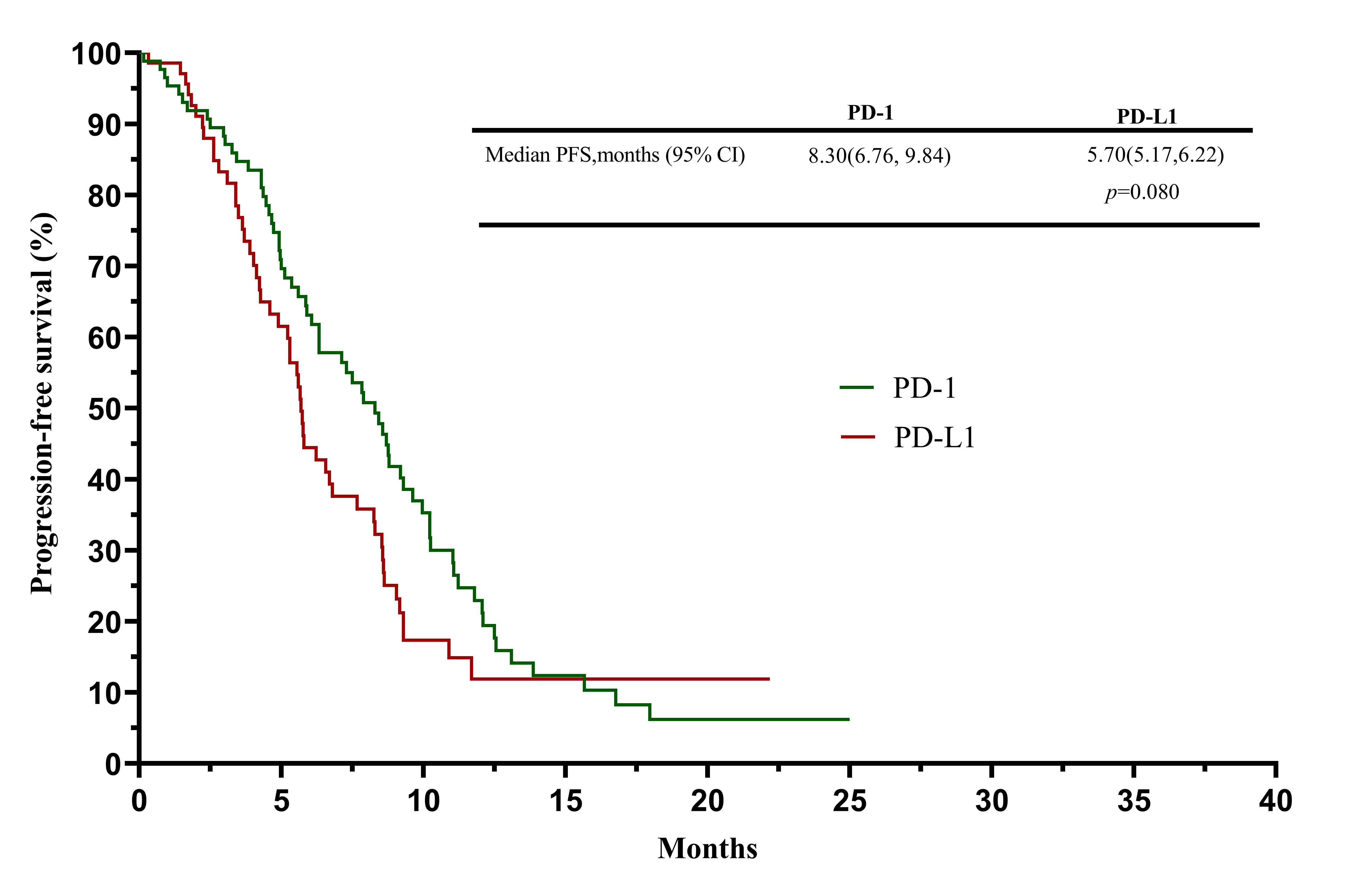


**Supplemental Figure 3.** Survival curves of progression-free survival (PFS) between extensive stage small cell lung cancer (ES-SCLC) patients who chose PD-1 inhibitors and PD-L1 inhibitors as the first-line (FL) treatment.


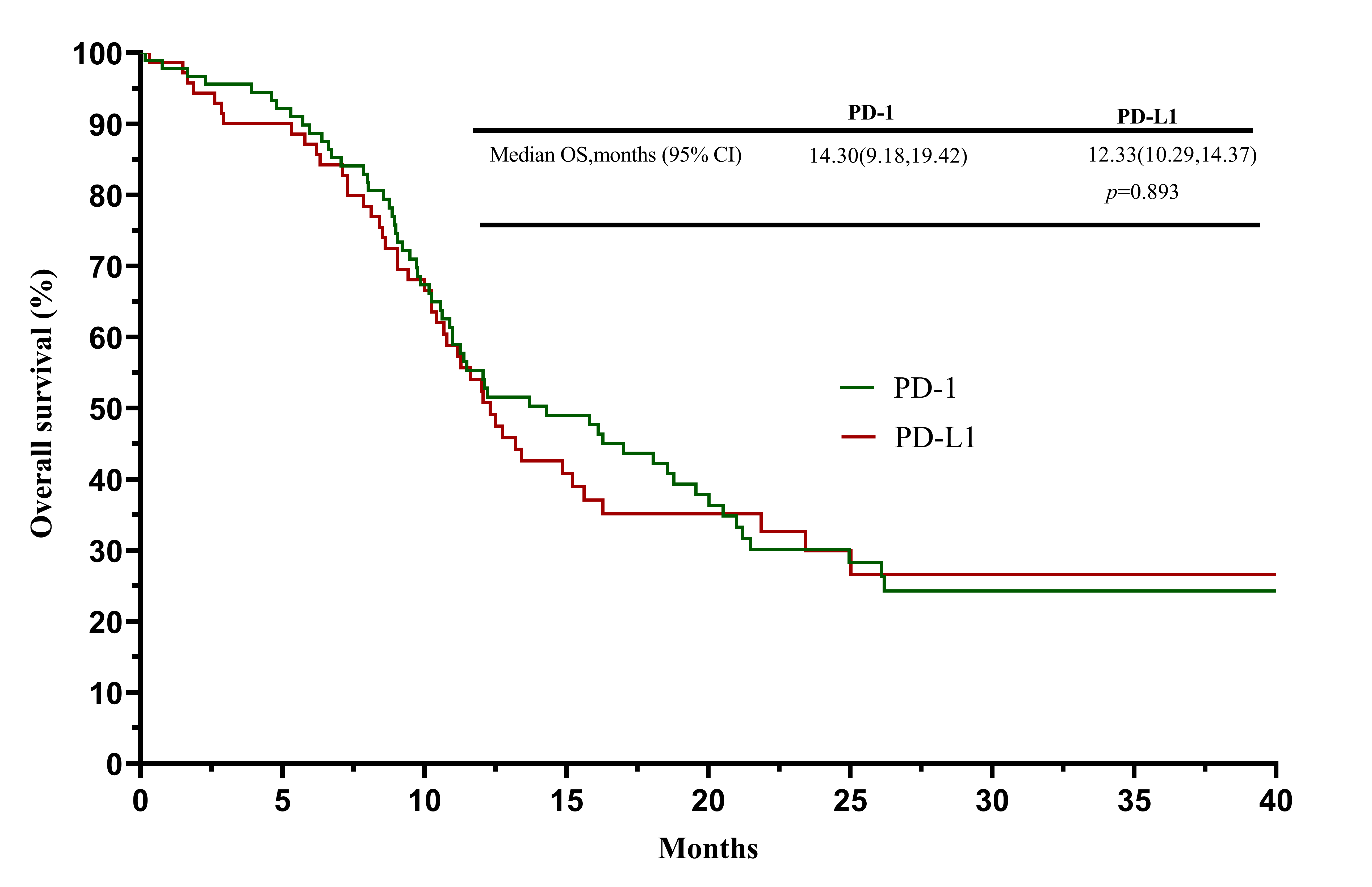


**Supplemental Figure 4.** Survival curves of overall survival (OS) between extensive stage small cell lung cancer (ES-SCLC) patients who chose PD-1 inhibitors and PD-L1 inhibitors as the first-line (FL) treatment.


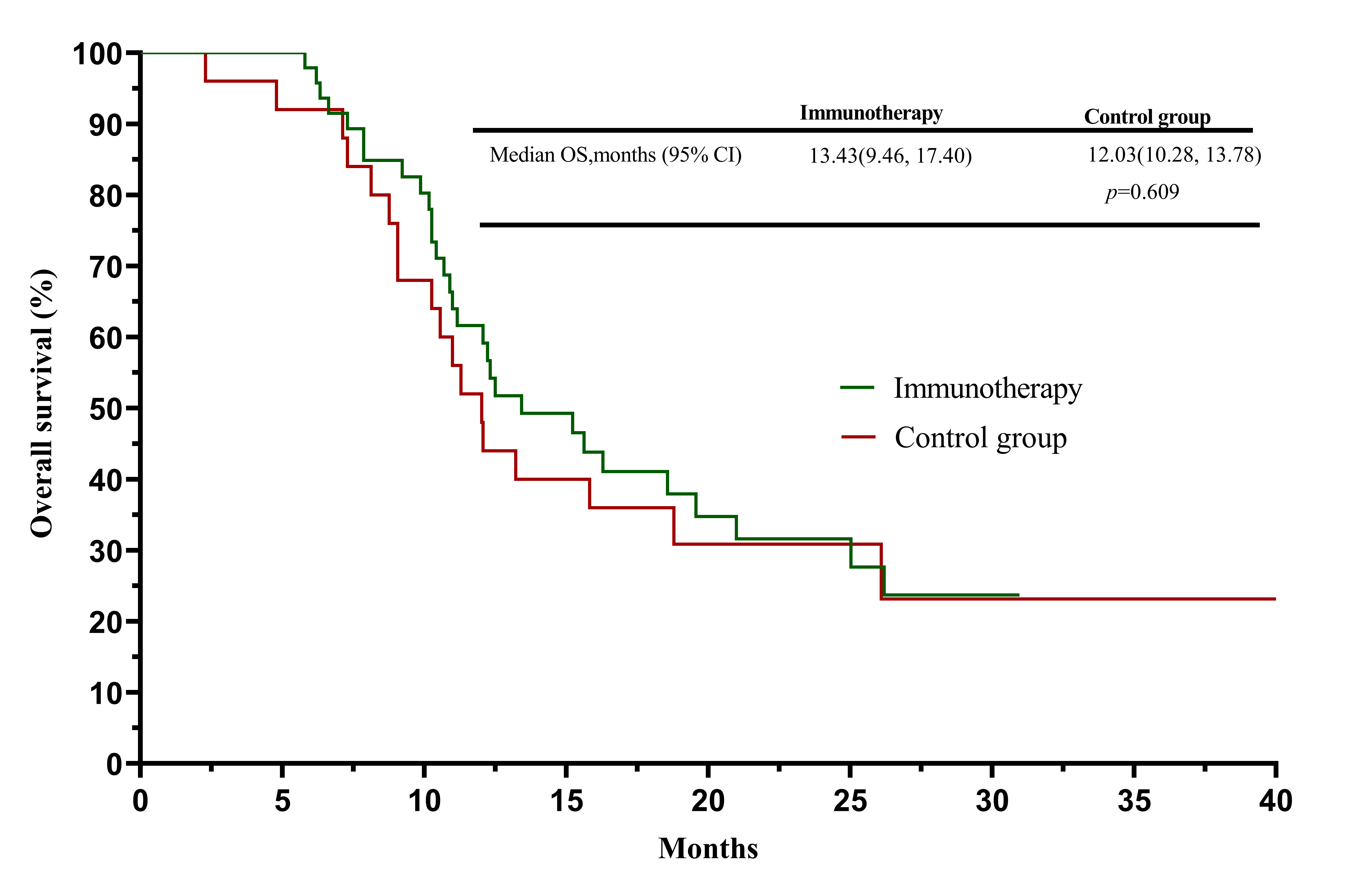


**Supplemental Figure 5.** Survival curves of overall survival (OS) between extensive stage small cell lung cancer(ES-SCLC) patients in chemo-immune group who chose immunotherapy and non-immunotherapy as the second-line treatment.
