# Supplementary material for: A retrospective real‐world experience of immunotherapy in patients with extensive stage small‐cell lung cancer
Source: Cancer Med. 2023 Jul 18;12(14):14881–91. doi: 10.1002/cam4.5843 (PMC10417203; doi:10.1002/cam4.5843)
Supplement: Supplementary file 2 — Table S1. Table S2. [file CAM4-12-14881-s001.docx]

**Supplemental Table 1. Treatment characteristics and prognosis of ES-SCLC patients between PD-1 inhibitors and PD-L1 inhibitors subgroups.**

| **Category and Subcategory** | **PD-1(n=91)** | **PD-L1(n=74)** | ***p* value** |
| --- | --- | --- | --- |
| **Response at the first evaluation, n** | | | |
| CR | 3 | 2 |  |
| PR | 61 | 51 |  |
| SD | 9 | 10 |  |
| PD | 4 | 6 |  |
| Not assessable | 14 | 5 |  |
| ORR (%) | 83.12 | 76.81 | 0.531 |
| DCR (%) | 94.81 | 91.30 | 0.517 |
| **irAE(3-4), n(%)**  Hypothyroidism  Pneumonia  Enteritis  Dermatitis  Hepatitis  Myocarditis  Thrombocytopenia | 11(52.38)  5  6  0  0  0  0  0 | 10(47.62)  0  2  2  0  2  1  1 | 0.785 |
| **Survival time(months)** |  |  |  |
| PFS | 8.30 | 5.70 | 0.080 |
| OS | 14.30 | 12.33 | 0.893 |

Abbreviation: ES-SCLC-extensive stage small cell lung cancer, CR-complete response, PR-partial response, SD-stable disease, PD-progressive disease, ORR-objective response rate, DCR-disease control rate, AEs-adverse events, irAEs-immune-related adverse events, ICIs-immune checkpoint inhibitors, PFS-progression-free survival, OS-overall survival.

**Supplemental Table 2. Clinical characteristics between the patients with immunotherapy and the control group in second-line treatment.**

| **Category and Subcategory** | **Total patients**  **(n=81)** | **Immunotherapy**  **(n=31)** | **Control (n=50)** | ***p* value** |
| --- | --- | --- | --- | --- |
| **Age (y)** | 61.53± 7.41 | 63.16± 8.48 | 60.52± 6.54 | 0.119 |
| **Gender, Male, n(%)** | 77(95.06) | 28(90.32) | 49(98.00) | 0.307 |
| **Smoking history, n(%)** | 59(72.84) | 23(74.19) | 36(72.00) | 0.829 |
| **PS scores, 0-1, n(%)** | 77(95.06) | 29(93.55) | 48(96.00) | 1.000 |
| **Location, Central, n(%)** | 66(81.48) | 25(80.65) | 41(82.00) | 0.879 |
| **Metastatic sites, n(%)** |  |  |  |  |
| Intrapulmonary | 14(17.28) | 7(22.58) | 7(14.00) | 0.321 |
| Liver | 10(12.35) | 6(19.35) | 4(8.00) | 0.245 |
| Brain | 21(25.93) | 7(22.58) | 14(28.00) | 0.589 |
| Bone | 16(19.75) | 6(19.35) | 10(20.00) | 0.943 |
| **COPD, n(%)** | 39(48.15) | 13(41.94) | 26(52.00) | 0.378 |
| **Interstitial pneumonia, n(%)** | 4(4.94) | 1(3.23) | 3(6.00) | 0.974 |
| **Second-line treatment, n(%)** |  |  |  |  |
| ICIs | 13(16.05) | 13(41.94) | 0(0.00) |  |
| ICIs+ chemotherapy | 18(22.22) | 18(58.06) | 0(0.00) |  |
| Chemotherapy | 50(61.73) | 0(0.00) | 50(100.00) |  |

Abbreviation: PS-performance status, COPD-chronic obstructive pulmonary disease, ICIs-immune checkpoint inhibitors.
